# Temporal Lobe Epilepsy Alters Auditory-motor Integration For Voice Control

**DOI:** 10.1038/srep28909

**Published:** 2016-06-30

**Authors:** Weifeng Li, Ziyi Chen, Nan Yan, Jeffery A. Jones, Zhiqiang Guo, Xiyan Huang, Shaozhen Chen, Peng Liu, Hanjun Liu

**Affiliations:** 1 Department of Rehabilitation Medicine, The First Affiliated Hospital, Sun Yat-sen University, Guangzhou, 510080, China; 2 Department of Neurology, The First Affiliated Hospital, Sun Yat-sen University, Guangzhou, 510080, China; 3 Ambient Intelligence and Multimodal Systems Lab, Shenzhen Institutes of Advanced Technology, Chinese Academy of Sciences Shenzhen, 518055, China; 4 Psychology Department and Laurier Centre for Cognitive Neuroscience, Wilfrid Laurier University, Waterloo, Ontario, N2L 3C5, Canada; 5 Department of Biomedical Engineering, School of Engineering, Sun Yat-sen University, Guangzhou, 510006, China; 6 Guangdong Provincial Key Laboratory of Brain Function and Disease, Zhongshan School of Medicine, Sun Yat-sen University, Guangzhou, 510080, China

## Abstract

Temporal lobe epilepsy (TLE) is the most common drug-refractory focal epilepsy in adults. Previous research has shown that patients with TLE exhibit decreased performance in listening to speech sounds and deficits in the cortical processing of auditory information. Whether TLE compromises auditory-motor integration for voice control, however, remains largely unknown. To address this question, event-related potentials (ERPs) and vocal responses to vocal pitch errors (1/2 or 2 semitones upward) heard in auditory feedback were compared across 28 patients with TLE and 28 healthy controls. Patients with TLE produced significantly larger vocal responses but smaller P2 responses than healthy controls. Moreover, patients with TLE exhibited a positive correlation between vocal response magnitude and baseline voice variability and a negative correlation between P2 amplitude and disease duration. Graphical network analyses revealed a disrupted neuronal network for patients with TLE with a significant increase of clustering coefficients and path lengths as compared to healthy controls. These findings provide strong evidence that TLE is associated with an atypical integration of the auditory and motor systems for vocal pitch regulation, and that the functional networks that support the auditory-motor processing of pitch feedback errors differ between patients with TLE and healthy controls.

Temporal lobe epilepsy (TLE) is the most common type of drug-refractory focal epilepsy, and is characterized by sclerosis in the mesial temporal regions^1^. Considerable evidence has shown that structural abnormalities in TLE, measured by volumetry, voxel-based morphometry, and cortical thickness, extend to fronto-central and parietal regions^2^,^3^,^4^. For example, TLE is associated with a significant reduction in the volume of the thalamus, cerebral hemispheres, and cerebellum^3^. Therefore, TLE is generally thought to be a system disorder because of the widespread nature of structural damage.

A growing body of literature has also shown patients with TLE exhibit disruptions in functional connectivity within certain networks^5^,^6^,^7^,^8^,^9^,^10^,^11^. For example, Bettus *et al*.^7^ found decreased functional connectivity in an epileptogenic network within the temporal lobes with concomitant contralateral increased connectivity. Compared to controls, patients with TLE exhibited significantly increased connectivity within the medial temporal lobes and significantly decreased connectivity within the frontal and parietal lobes, and between frontal and parietal lobes^6^. Graph-theoretical analyses have shown that patients with TLE exhibit topological alterations in the functional networks toward a progressively more random network^5^,^6^ or a more regularized network^11^,^12^ as compared to healthy controls. Moreover, longer durations of TLE are associated with lower functional connectivity and more random neural network configurations^5^,^6^,^10^, suggesting a progressive reorganization of large-scale interregional functional networks in TLE.

The extensive nature of these changes in the structural and functional brain networks has a negative impact on the cognitive functions of patients with TLE, such as problems with perceptual organization, academic achievement, language, and visuospatial function^13^. In the auditory domain, patients with TLE exhibit decreased performance in temporal ordering and dichotic listening tasks when listening to both verbal and nonverbal sounds^14^, deficits in processing rapid sequential auditory information^15^, and increased amplitudes and/or latencies of mismatch negativity (MMN) in response to deviant tones^16^,^17^,^18^. Moreover, across a number of auditory tasks, patients with TLE have been found to exhibit a significant positive correlation between the latencies of auditory cortical responses and the duration of the disease^19^,^20^. These studies are suggestive of impaired central auditory processing in patients with TLE. Whether those behaviors that require the involvement of auditory function, such as speech motor control, are compromised by TLE, however, is largely unknown.

Speech motor control relies on sensory feedback, particularly auditory feedback, while speakers learn to produce new sounds, as well as to detect errors in vocal productions^21^,^22^. Current theories and computational models that hypothesize the neural mechanisms of speech motor control^23^,^24^,^25^ all describe a process whereby “efference copies” of motor commands are used to generate predictions of auditory (and kinesthetic) feedback, which are compared to the incoming auditory feedback. When discrepancies are detected between auditory feedback and the expected auditory feedback, corrective motor commands are initiated that compensate for the perceived vocal error.

To understand these hypothesized mechanisms, researches have exposed speakers to altered auditory feedback during vocal production and measured behavioral and brain responses to the perturbations. For example, when speakers hear frequency-altered feedback (FAF), they tend to rapidly adjust their vocal pitch in the opposite direction of the perturbation^26^,^27^. These behavioral changes are paralleled by modulations in event-related potential (ERP) components of the N1-P2 complex^28^,^29^,^30^. Recently, studies using neuroimaging techniques (e.g. fMRI, MEG) implicate brain regions that include the superior temporal gyrus (STG), posterior superior temporal sulcus (pSTS), anterior cingulate cortex (ACC), dorsal premotor cortex (dPMC), inferior frontal gyrus (IFG), and inferior parietal lobe (IPL), in planning and execution of the vocal compensation for pitch perturbations^31^,^32^,^33^,^34^.

Despite the advanced progress in understanding the neural mechanisms of speech motor control, much less is known about auditory-motor integration for voice control in TLE. Electrocorticography (ECoG) performed on drug-refractory epilepsy patients during the interictal phase has recently received significant attention from researchers investigating auditory-motor integration for voice control^35^,^36^,^37^ because ECoG affords high spatio-temporal resolution and excellent signal-to-noise properties. For example, both Chang *et al*.^35^ and Greenlee *et al*.^36^ found increased high-gamma brain activity in the STG in response to perturbations heard in vocal pitch feedback compared to when participants passively listened to recordings of their own speech. Moreover, increased brain activity began in the posterior STG and was followed by increased activity in the ventral PMC, and activity in these two brain regions was correlated positively with the magnitude of vocal compensation on a trial-by-trial basis^35^.

Although these ECoG studies shed light on the neural mechanisms that underlie sensorimotor control of vocal production, it is at present unknown whether TLE compromises the integration of auditory information into the vocal motor system during vocalization. Since intra-cranial signals are only recorded from epilepsy patients to localize epileptogenic brain regions, there is no direct evidence that auditory-motor integration in TLE patients differs from healthy controls. However, previous research has shown that individuals with Parkinson’s disease (PD), who often have deficits in perceiving self-produced speech^38^ and disruptions in the functional connectivity within the fronto-temporal and parietal networks^39^,^40^, are impaired in the sensorimotor control of vocal production as reflected by their significantly larger vocal compensation for pitch feedback errors than healthy controls^41^,^42^. It is thus possible that patients with TLE also have atypical integration of auditory feedback into the vocal motor system during vocalization due to their central auditory processing disorders and structural and functional abnormalities in the fronto-temporal and parietal networks.

Therefore, the present ERP study was designed to examine whether patients with TLE differ from healthy controls in the auditory-motor processing of vocal pitch regulation behaviorally and neurally. Both patients with TLE and healthy controls were exposed to FAF during vocalization, and vocal and ERP responses were measured and compared across conditions. Furthermore, we used the graph-theoretical method to analyze the ERP responses to FAF in the theta band to investigate whether the functional network configuration that supports auditory-motor integration in patients with TLE differed from the network configuration observed in healthy controls. The graph-theoretical method offers a formal framework to quantify the topological and organizational properties of complex interconnected networks^11^,^43^,^44^. We expected that vocal and ERP responses to perceived vocal errors in auditory feedback observed in patients with TLE and healthy controls would differ significantly, and that these neurobehavioral differences would be likewise demonstrated by differences in their function network configuration. Finally, we hypothesized that there would be a relationship between the duration of the disease and the pattern of vocal and/or ERP responses observed in patients with TLE.

## Results

### Vocal responses

**Figure 1:**
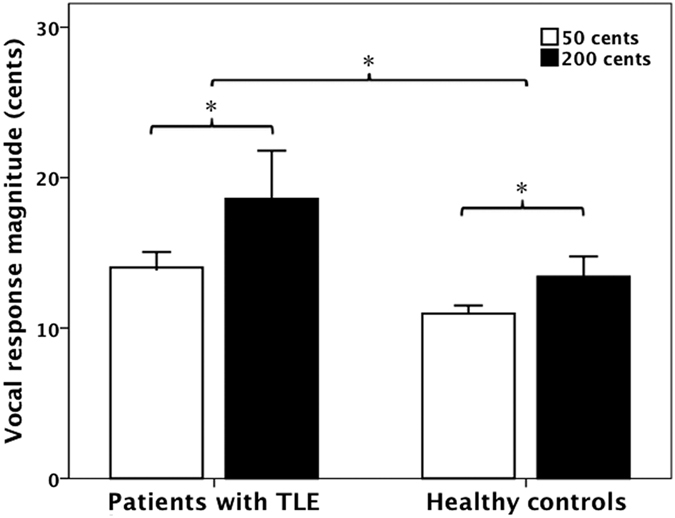
T-bar plots of the magnitudes (means and standard errors) of vocal responses produced by patients with TLE and healthy controls to +50 cents (blank bars) and +200 cents (black bars) pitch shifts. Patients with TLE produced significantly larger response magnitudes than healthy controls (F(1, 53) = 6.020, p = 0.017), and pitch shifts of +50 cents elicited significantly smaller response magnitudes than pitch shifts of +200 cents (F(1, 53) = 10.428, p = 0.002).

Figure 1 shows T-bar plots of the vocal response magnitudes as a function of stimulus and group. Since the vocal responses produced by one patient with TLE did not meet our criteria for valid responses, only the vocal response data of 27 patients with TLE were statistically analyzed. A two-way ANOVA conducted on the response magnitudes revealed that patients with TLE (16.3 ± 12.4 cents) produced significantly larger response magnitudes than healthy controls (12.2 ± 5.5 cents) (F(1, 53) = 6.020, p = 0.017), and pitch shifts of +50 cents (12.5 ± 4 cents) elicited significantly smaller response magnitudes than pitch shifts of +200 cents (16.0 ± 12.8 cents) (F(1, 53) = 10.428, p = 0.002). No significant difference was found in the interaction between stimulus magnitude and group (F(1, 53) = 0.047, p = 0.829). As for the response latencies, the main effects of stimulus magnitude (F(1, 53) = 0.067, p = 0.796) and group (F(1, 53) = 1.889, p = 0.175) as well as interactions between stimulus and group (F(1, 53) = 0.581, p = 0.449) did not reach significance.

**Figure 2:**
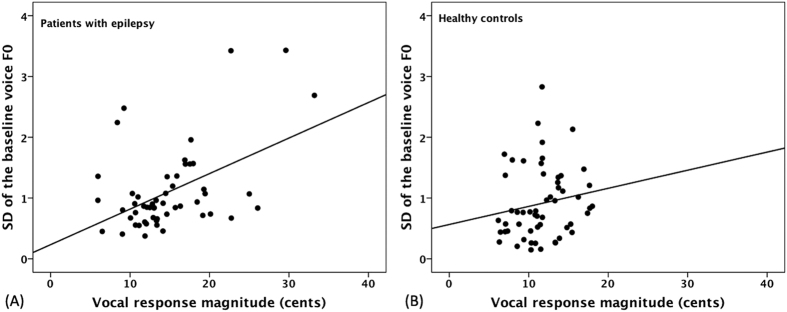
Correlations between the SDs of the baseline mean F_0_ and the magnitude of vocal responses for patients with TLE (**A**) (p < 0.001, *r* = 0.48) and healthy controls (**B**) (p = 0.420, *r* = 0.11).

Regression analyses were performed to examine the relationship between the variability of the baseline voice F_0_ and the magnitude of vocal responses to pitch perturbations. The magnitudes of vocal responses are plotted against the standard deviations (SDs) of the baseline voice F_0_ for patients with TLE and healthy controls in Fig. 2. As can be seen, patients with TLE exhibited a significant positive correlation between the SDs of the baseline voice F_0_ and the magnitudes of vocal responses (p < 0.001, *r* = 0.48), indicating that variability of the baseline voice F_0_ was predicative of the degree of vocal compensation for pitch feedback errors. By contrast, this correlation did not reach significance for healthy controls (p = 0.420, *r* = 0.11).

### ERP responses

**Figure 3:**
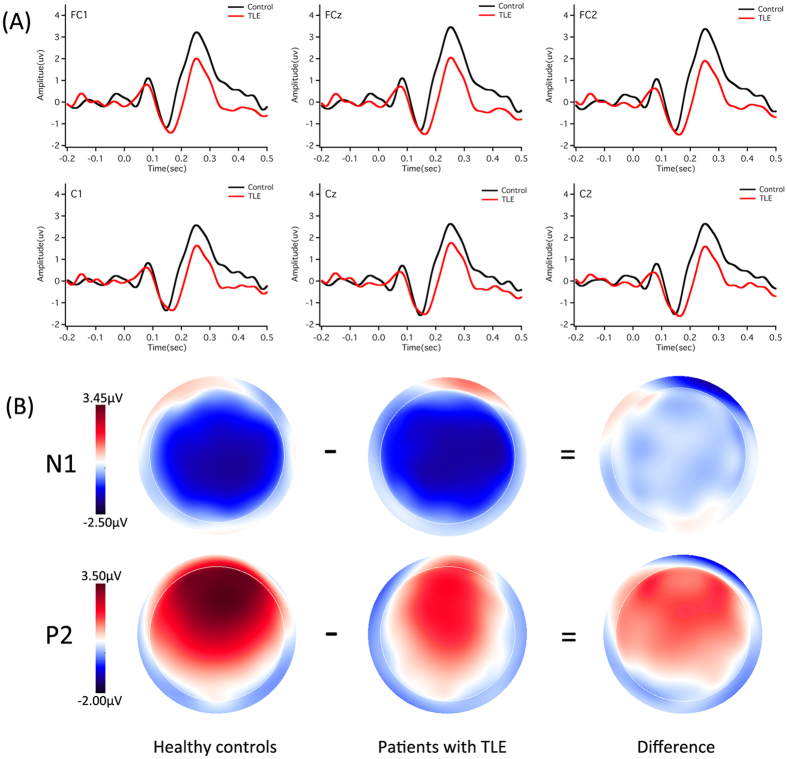
Grand-averaged ERP waveforms (**A**) and topographical distributions of N1 and P2 amplitudes (**B**) in response to pitch shifts of +50 cents. The red and black solid lines denote the cortical responses produced by patients with TLE and healthy controls, respectively.

**Figure 4:**
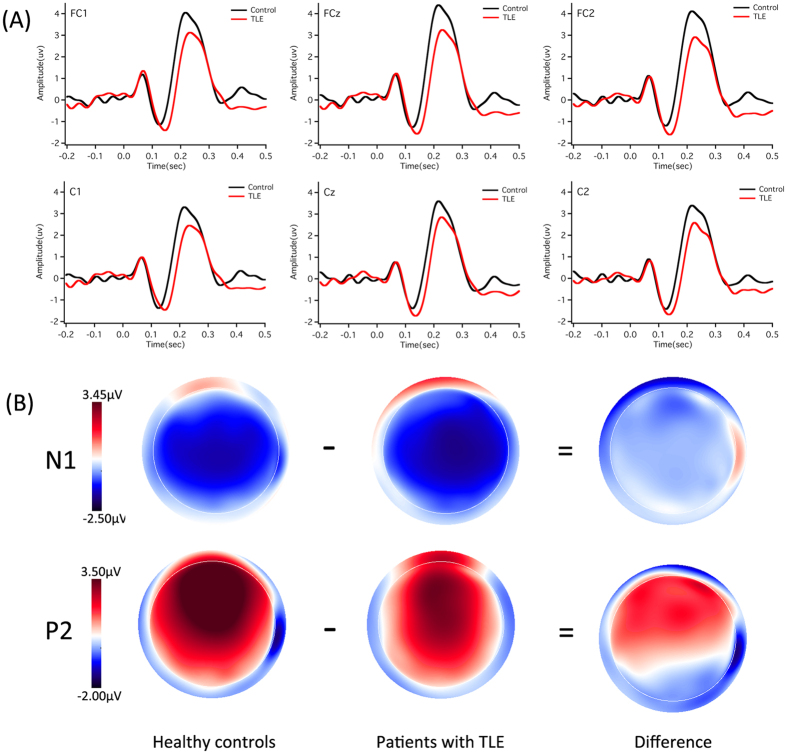
Grand-averaged ERP waveforms (**A**) and topographical distributions of N1 and P2 amplitudes (**B**) in response to pitch shifts of +200 cents. The red and black solid lines denote the cortical responses produced by patients with TLE and healthy controls, respectively.

Figures 3 and 4 show grand-averaged ERP waveforms (A) and topographical distributions of N1 and P2 amplitudes (B) in response to pitch shifts of +50 and +200 cents produced by patients with TLE (red solid lines) and healthy controls (black solid lines). As can be seen, patients with TLE produced smaller P2 responses to both +50 and +200 cents pitch shifts than healthy controls. N1 responses, however, appeared to be similar between two groups. This group difference can be further observed in the topographical distributions of the N1-P2 complex.

A three-way ANOVA conducted on the N1 amplitudes revealed no significant main effect of stimulus magnitude (F(1, 54) = 0.010, p = 0.920) or group (F(1, 54) = 1.043, p = 0.312). There was a significant main effect of electrode site (F(9, 486) = 6.684, p < 0.001), however, which was mainly caused by less negative N1 amplitudes associated with FC3 and C3 when compared with N1 amplitudes recorded from FC4 and C2. No interactions between these factors reached significance (p > 0.05).

Regarding the N1 latencies, there was a significant main effect of stimulus magnitude (F(1, 54) = 21.121, p < 0.001), indicating that pitch shifts of +50 cents elicited significantly longer N1 latencies than pitch shifts of +200 cents (145 ms vs. 129 ms). The main effects of electrode site (F(9, 486) = 1.041, p = 0.372) and group (F(1, 54) = 0.614, p = 0.437), however, did not reach significance. No significant interaction between any of these variables was found either (p > 0.05).

**Figure 5:**
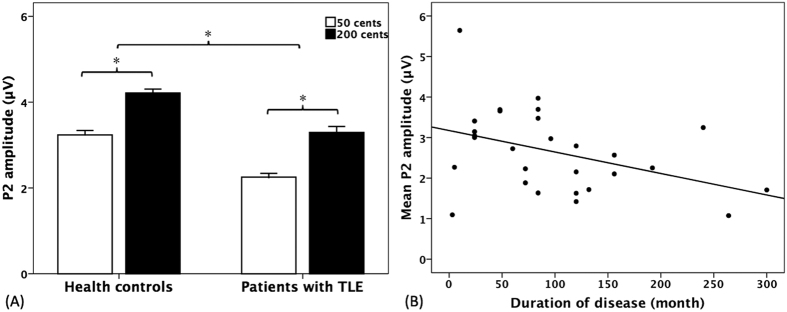
(**A**) T-bar plots of P2 amplitudes (means and standard errors) in response to +50 cents (blank bars) and +200 cents (black bars) produced by patients with TLE and healthy controls. Patients with TLE produced significantly smaller P2 amplitudes than healthy controls (F(1, 54) = 8.883, p = 0.004), and pitch shifts of + 50 cents elicited significantly smaller P2 amplitudes than pitch shifts of + 200 cents (F(1, 54) = 13.131, p = 0.001). (**B**) Scatter plots with trend line showing the mean amplitudes of P2 response to pitch feedback perturbations as a function of the duration of epilepsy disease (p = 0.033, *r* = −0.404).

A three-way ANOVA conducted on the P2 amplitudes revealed that patients with TLE produced significantly smaller P2 amplitudes than healthy controls (F(1, 54) = 8.883, p = 0.004), and pitch shifts of +50 cents elicited significantly smaller P2 amplitudes than pitch shifts of +200 cents (F(1, 54) = 13.131, p = 0.001) (see Fig. 5A). There was also a significant main effect of electrode site (F(9, 486) = 43.192, p < 0.001), which was primarily driven by larger P2 amplitudes associated with central electrodes as compared to lateral electrodes. However, no interactions between any of these variables reached significance (p > 0.05).

Regarding the P2 latencies, there was a significant main effect of stimulus magnitude (F(1, 54) = 26.699, p < 0.001), indicating that pitch shifts of +50 cents elicited significantly longer P2 latencies than pitch shifts of +200 cents (256 ms vs. 234 ms). The main effects of site (F(9, 486) = 3.883, p = 0.054) and group (F(1, 54) = 1.381, p = 0.245), however, did not reach significance. As well, there were no interactions between these variables (p > 0.05).

We also investigated whether vocal and cortical responses to pitch perturbations were affected by disease progress. The mean amplitudes of P2 responses across conditions are plotted against the disease duration in Fig. 5B. The results revealed that the mean P2 amplitudes were significantly correlated with the disease duration (p = 0.033, *r* = −0.404), indicating that P2 amplitudes decreased with increased duration of the disease. The duration of epilepsy, however, was not correlated with N1 amplitudes (p = 0.746, *r* = −0.064) or vocal response magnitudes (p = 0.960, *r* = 0.010).

### Graphical network results

**Figure 6:**
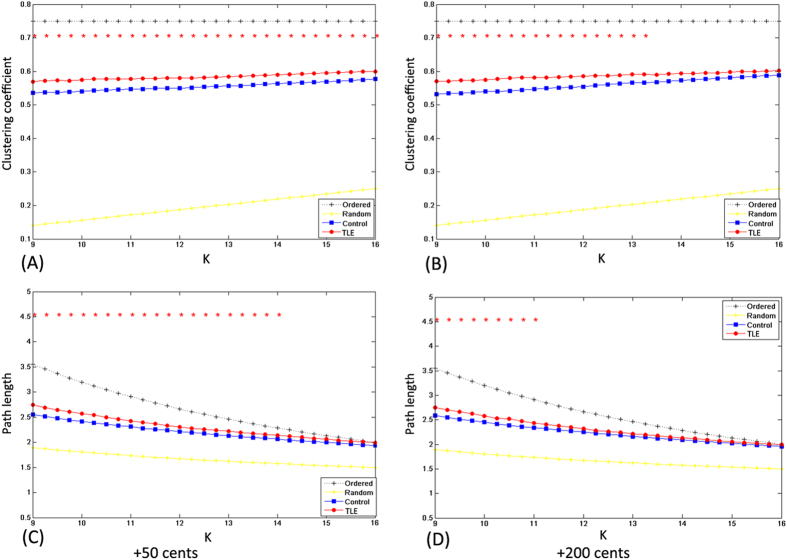
Top: mean clustering coefficients in the case of + 50 cents (**A**) and +200 cents (**B**) for patients with TLE (red dots) and healthy controls (blue squares) as a function of *K*. Bottom: mean absolute path lengths in the case of +50 cents (**C**) and +200 cents (**D**) for patients with TLE (red dots) and healthy controls (blue squares) as function of *K*. Red asterisks indicate where the difference between the two groups is significant (*t*-test, p < 0.05). The theoretical values of *C* and *L* for ordered and random networks as a function of *K* are shown for comparison.

**Figure 7:**
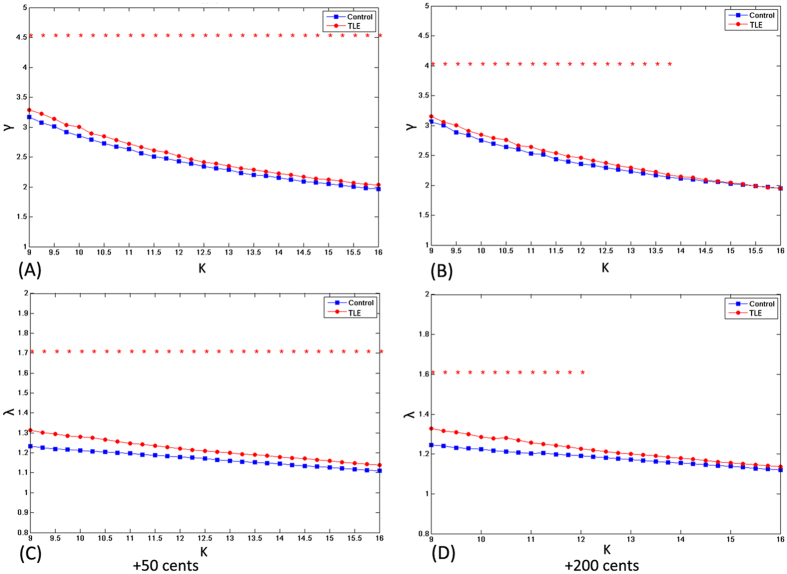
Top *γ* in the case of +50 cents (**A**) and +200 cents (**B**) for patients with TLE (red dots) and healthy controls (blue squares). Bottom: *λ* in the case of +50 cents (**C**) and +200 cents (**D**) for patients with TLE (red dots) and healthy controls (blue squares). Red asterisks indicate where the difference between the two groups is significant (*t*-test, p < 0.05).

Figures 6 and 7 show the network parameters of clustering coefficient *C*, absolute path length *L*, λ, and *γ* as a function of degree *K* for both patients with TLE and healthy controls in the +50 and +200 cents conditions. As compared to healthy controls, patients with TLE exhibited a significant increase of clustering coefficient *C* over a wide range of *K* for both +50 and +200 cents pitch shifts (all p < 0.05). Similarly, the absolute path length *L* showed a significant increase for patients with TLE (p < 0.05) over a wide range of *K* (9 ≤ *K* ≤ 14) in the case of +50 cents pitch shifts, but over a narrow range of *K* (9 ≤ *K* ≤ 11) in the case of +200 cents pitch shifts. Figure 7 shows a small-world organization in both patients with TLE and healthy controls, which is reflected in 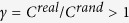 and 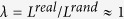 over the whole range of degree *K*. Also, we observed significantly larger λ and *γ* for the patients with TLE as compared to healthy controls over the whole range of degree *K* in the +50 cents condition. This group difference was also observed in the +200 cents condition for low values of degree *K*. In summary, significant differences between the two groups in the network parameters indicate a disrupted functional network regulates the auditory-motor processing of pitch feedback errors in patients with TLE.

## Discussion

The present study investigated whether patients with TLE exhibited atypical auditory-motor integration for voice control. As expected, when exposed to FAF, patients with TLE exhibited patterns of behavioral responses and cortical activity that differed from healthy controls in several ways. First, patients with TLE produced larger vocal responses than healthy controls and exhibited a positive correlation between the magnitudes of their vocal responses and the SDs of their baseline voice F_0_. Second, patients with TLE produced smaller P2 responses than healthy controls and showed a negative correlation between P2 amplitudes and epilepsy duration. Finally, the functional neural network that supports auditory-motor processing of pitch feedback errors in patients with TLE appeared disrupted, as reflected by significantly higher clustering coefficients and longer absolute path lengths than healthy controls. These findings provide the first evidence that patients with TLE present atypical auditory-motor integration for vocal pitch regulation, suggesting that TLE is associated with a progressive functional decline of the sensorimotor systems that underlie vocal pitch monitoring.

### Behavioral findings

In the present study, patients with TLE produced larger vocal responses to FAF than healthy controls. Although other studies have reported vocal responses to pitch feedback perturbations produced by epilepsy patients, prior to or undergoing ECoG monitoring^35^,^36^,^45^, they differed in their methods in several ways. For example, Chang *et al*.^35^ measured the trial-to-trial vocal responses to FAF of −200 cents obtained from 7 epilepsy patients, while Greenlee *et al*.^36^ and Behroozmand *et al*.^45^ separately measured the mean vocal responses to FAF of −100 cents from 8 epilepsy patients, and FAF of +600 cents from 8 epilepsy patients, respectively. In addition, patients with other types of epilepsy such as frontal lobe epilepsy were also included as participants in addition to TLE. Although differences in the methodology make direct compared difficult, one commonality across these studies is the relatively small number of participants. These power issues are of course unavoidable in this patient population, and just as unavoidable is that none of these studies could include healthy controls, at least in terms of recording ECoG, and thus were unable to determine whether epilepsy patients differed from healthy populations in their vocal and neural responses to FAF. In contrast, the present findings provide evidence for the first time that patients with TLE significantly differ from healthy controls in their rapid vocal adjustment for unexpected pitch feedback perturbations.

Enhanced vocal responses to FAF observed for patients with TLE reflect their atypical auditory-motor integration for voice control, which may be due to dysfunction in the feedback-based perception and/or dysfunction in the motor system responsible for execution of vocal pitch corrections. Previous research has shown that patients with TLE have deficits in the auditory processing of speech and non-speech sounds during temporal ordering and dichotic listening tasks^14^,^15^,^46^. On the other hand, speech motor control involves a weighting of feedback and feedforward control systems, and over-reliance on feedback control leads to errors in speech production^47^. A significant positive correlation between the magnitude of vocal responses and the variability of the baseline voice F_0_ observed for patients with TLE suggests that the increased motor variability experienced by patients with TLE during their day-to-day vowel productions induces a bias towards more feedback guided vocal production. That is, patients with TLE may weight the discrepancy between incoming auditory feedback and their expected feedback more heavily than feedforward input to the motor control system, which results in significantly larger vocal responses compared to healthy controls. By contrast, the absence of this correlation in healthy controls may indicate that their relatively less variable motor production permits greater reliance on feedforward input to the motor control system, and they are therefore less susceptible to alterations to their auditory feedback, and hence produce smaller vocal responses as compared to patients with TLE.

On the other hand, Chang *et al*.^35^ noted that the motor areas mediate the vocal responses to FAF via projections from the auditory areas and there is a positive correlation between the high-gamma activity in the motor cortex and the magnitude of vocal responses. By using transcranial magnetic stimulation (TMS), previous research has shown an increased cortical excitability of the motor cortex associated with TLE^48^,^49^. Thus, the larger vocal responses to FAF produced by patients with TLE could be caused by enhanced neuronal excitability of the motor cortex as compared to healthy controls.

### ERP findings

At the cortical level, patients with TLE produced significantly smaller P2 responses to FAF than did healthy controls. This finding is in line with previous studies that showed decreased P2 or M150 (the magnetic counterpart of the P2 component) response to auditory stimuli in patients with focal epilepsy^19^,^50^,^51^. An effect of epilepsy on the N1 response to FAF, however, was not found. Similarly, previous studies of auditory perception on patients with focal epilepsy have also showed an intact N1 response^50^,^52^. In addition, other studies have shown that patients with TLE differ significantly from healthy controls in auditory discrimination as reflected by attenuated^19^,^53^ or enhanced MMN^16^,^17^. These findings generally reflect a dysfunction of cortical auditory information processing in patients with TLE. Since P2 responses to FAF during vocal pitch regulation are thought to reflect general auditory processing, as well as more specific feedback-based motor processing^28^,^54^, our results further demonstrate that patients with TLE have impaired auditory-motor integration for voice control at the level of the cortex.

The hypothesis that auditory-motor integration is compromised in TLE is further supported by the finding of a significant negative correlation between epilepsy duration and P2 amplitudes in response to FAF: the longer duration of epilepsy disease appears to lead to a progressive modification of the cortical processing of feedback errors during vocal pitch regulation. This finding is complementary to other studies that show positive correlations between epilepsy duration and increased latencies of auditory cortical responses^19^,^20^. In addition, patients with TLE exhibit lower functional connectivity and more random network configuration associated with longer epilepsy duration^5^,^8^,^10^. Taken together with previous studies, the present study indicates that there is a functional decline of the neural networks that support auditory-motor processing of feedback errors in TLE, and this decline progresses with increasing duration of the disease.

Changes in the size of P2 responses may not only reflect the detection of errors in voice auditory feedback, but also the interaction between the auditory and motor systems^28^. There is evidence that regions in the Sylvian fissure may be a source of the P2 component^55^. The posterior Sylvian fissure (area Spt) has been hypothesized to perform a coordinate transformation between auditory and motor representations^56^. On the other hand, Zhang *et al*.^57^ reported decreased functional connectivity within the regions of the auditory and sensorimotor networks in TLE, including the bilateral superior temporal cortex, the precentral, postcentral, and medial frontral gyri, as well as the supplementary motor area. Most of these brain regions have been shown to be involved in the auditory-motor integration in voice control^33^,^45^. Furthermore, our graph-theoretical analyses revealed significantly longer absolute path lengths for patients with TLE relative to healthy controls. Short absolute path lengths are thought to promote effective interactions between and across cortical brain regions^58^. The longer absolute lengths in TLE are likely suggestive of decreased efficiency of parallel information transfer between interconnected brain regions in the auditory-motor processing of pitch feedback errors. In addition, seizure activities from the hippocampus can exert long-range effects on the sensorimotor cortex^59^. In light of these findings, epileptic activity may persistently affect the neural substrates involved in the auditory-motor integration for voice control, leading to the reduced or less effective interactions between different brain regions, or functional connectivity, within the sensorimotor network, which may in turn result in decreased P2 responses to FAF.

### Changes in network properties

The graph-theoretical analyses of the ERP responses to FAF in the theta band showed increased clustering coefficient *C* and absolute path length *L* over a wide range of network thresholds in patients with TLE as compared to healthy controls, indicating that patients with TLE have a disrupted topology of the brain functional networks that support the auditory-motor integration for voice control. Although patients with TLE also exhibited small-world topological characteristics (as confirmed by 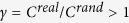 and 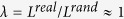 over the whole range of degree *K*), higher *C* and *L* values suggest subtle topological alterations in their functional networks toward a more regularized configuration as compared to healthy controls in the auditory-motor processing of pitch feedback errors. The direction of topological alterations in the functional networks observed in the present study closely resembles those found in the graph-theoretical analyses of the EEG or fMRI signals in the interictal phase^11^,^12^ or during focal seizures^60^,^61^. Another pattern of network disruptions in TLE, however, has been reported in other studies that patients with TLE exhibited a progressively more random network (i.e. low *C* and *L*)^5^,^6^. The divergence between the results of these studies likely stems from differences in the clinical inclusion criteria and experimental paradigms.

Short absolute path lengths in the brain assure effective interactions between and across different cortical regions, while high clustering coefficients reflect high local efficiency of information transfer and robustness^43^. As compared to healthy controls, longer absolute path lengths associated with TLE may indicate less efficient information interactions between interconnected brain regions, making signal propagation speed between temporolimbic and extratemporal neocortical networks slower^58^. Patients with TLE also exhibited increased clustering coefficients in the present study, indicating that they are associated with a stronger local specialization. Considering that the small-world brain favors a selection of maximizing cost efficiency of both local specialization and global interaction in large-scale networks, increased absolute path lengths and clustering coefficients associated with TLE may disrupt this optimal balance in information processing, leading to their atypical auditory-motor integration for voice control. It has been suggested that a more regularized network configuration is characteristic of reduced signal propagation speed and synchronizability^62^, and thus its resilience to pathological attacks is weakened^58^, which may contribute to the decline in various cognitive functions in TLE^63^. Overall, changes in small-world parameters observed in the present study reflect a less optimal topological organization in TLE, providing further evidence for a disrupted functional network topology in their auditory-motor processing of feedback errors during vocal pitch regulation.

### Limitations

Because most of the patients with TLE in the present study received multiple drug therapy, we cannot rule out the possibility that the observed changes in the auditory-motor integration for voice control could be partially due to the long-term effect of antiepileptic drugs (AEDs). However, there is evidence suggesting that most AEDs have little or no effect on cognitive function when they are used at recommended doses^64^ and the role of AEDs may be less important than that of the epilepsy itself^65^. Although AEDs have been shown to have effects on motor reaction times and ERP response latencies^66^,^67^, we did not observe any differences between patients with TLE and healthy controls in the latencies of either vocal or cortical responses to FAF in the present study. As well, because patients with TLE were off medication for the 12 hours prior to experimental session, the acute effect of AEDs on the neurobehavioral responses to FAF in the present study are likely minimal. Larger-scale studies, however, are still needed to determine the effects of AEDs on the auditory-motor integration in voice control.

## Conclusion

The present study examined whether patients with TLE had atypical auditory-integration for voice control. The results revealed that, as compared to healthy controls, patients with TLE produced significantly larger vocal responses and smaller cortical responses to FAF during vocal pitch regulation and exhibited a significant negative correlation between P2 responses and epilepsy duration. Moreover, patients with TLE had increased clustering coefficients and absolute path lengths, indicating for the first time that the brain networks in patients with TLE are less small-world like than the brain networks of healthy controls. Taken together, these findings demonstrate that patients with TLE differ significantly from healthy populations in their auditory-motor integration for voice control.

## Materials and Methods

### Subjects

Twenty-eight patients with TLE (10 females and 18 males; mean age = 27.32 ± 5.96 years) were recruited from the Department of Neurology at The First Affiliated Hospital of Sun Yat-sen University in China. All patients had been diagnosed with TLE on the basis of a clinical history of seizures, MRI, CT, and EEG, and have been prescribed one to three appropriate AEDs. Eligibility criteria included the following: (1) Full Scale IQ > 80 and no apparent intellectual disability, or attention disorders prior to onset of seizure; (2) no history of temporal lobectomy; (3) epileptic spikes in the bilateral frontotemporal or temporal lobes. MRI was performed for all 28 patients and showed hippocampal sclerosis/atrophy without extratemporal lesions in 19 patients and normal findings in 9 patients. Finally, 17 patients were diagnosed as having right-sided TLE and 11 as having left-sided TLE. Twenty-eight healthy participants (10 females and 18 males; mean age = 25.79 ± 5.92 years) with normal IQ were recruited as controls. Both patients with TLE and healthy controls were right-handed, native-Mandarin speakers, and none of them had a history of speech, hearing, language, or neurological disorders. All participants passed a hearing screening at 25 dB hearing level (HL) for octave intervals of 500–4000 Hz.

Informed consent was obtained from all participants. All the procedures, including subject recruitment and data acquisition, were approved by the Institutional Review Board of The First Affiliated Hospital at Sun Yat-sen University of China in accordance with the Code of Ethics of the World Medical Association (Declaration of Helsinki).

### Apparatus

The experiment was carried out in a sound-treated booth. In order to partially mask air-born and bone-conducted voice feedback, we calibrated the experimental system to ensure that participants heard their voice feedback with a gain of 10 dB sound pressure level (SPL) relative to the intensity of their vocal output. The voice signals were transduced by a dynamic microphone (model DM2200, Takstar Inc.), amplified by a MOTU Ultralite Mk3 firewire audio interface, and pitch-shifted by an Eventide Eclipse Harmonizer in real time. A custom-developed MIDI program (Max/MSP, v.5.0 by Cycling 74) running on a Macintosh computer was used to control the Harmonizer to shift the voice pitch feedback. This program also generated transistor-transistor logic (TTL) pulses to mark the onset of each pitch perturbation. The TTL pulses were also sent to the EEG recording system via a synch DIN cable. Finally, the voice signals were amplified by an ICON NeoAmp headphone amplifier and fed back to participants through insert earphones (ER1-14 A, Etymotic Research Inc.). The original and pitch-shifted voice signals as well as the TTL pulses were digitized with a sampling frequency of 10 kHz by a PowerLab A/D converter (model ML880, AD Instruments), and recorded onto another Macintosh computer using LabChart software (v.7.0 by AD Instruments).

### Procedure

Participants were instructed to produce sustained phonation of the vowel /u/ for approximately 5–6 seconds at their conversational pitch and loudness level. During each vocalization, they heard their voice unexpectedly pitch-shifted upwards by 50 or 200 cents (100 cents = 1 semitone). The size of pitch shifts was manipulated because previous research has shown that the magnitude of the feedback perturbations affects both the vocal and ERP responses to pitch-shifted auditory feedback^28^. Participants were required to take a 2–3 s break between successive vocalizations to avoid the vocal fatigue. During each vocalization, 5 pitch shifts (200 ms duration) were presented with an inter-stimulus interval of 700–900 ms. The first stimulus occurred 500–1000 ms after the vocal onset. Participants produced 40 consecutive vocalizations, resulting in 100 + 50-cent trials and 100 + 200-cent trials.

### Behavioral Data analyses

Vocal responses to pitch-shifted auditory feedback were measured using a custom program created with IGOR PRO (v.6.0, Wavemetrics Inc.). First, voice F_0_ contours in Hertz were extracted from the voice signals in Praat^68^, and converted to a cent wave using the following formula: cents = 100 × (12 × log_2_(F_0_/reference)). The reference denotes an arbitrary reference note of 195.997 Hz (G4). All trials were segmented into epochs ranging from −200 ms to 700 ms relative to the onset of the pitch shift. Based on visual inspection, trials containing vocal interruption or signal processing errors were excluded from further analyses. Finally, the artifact-free trials were averaged to generate an overall response for each condition. A valid vocal response was defined as the averaged cent waveform exceeding 2 SDs of the baseline mean F_0_ that occurred at least 60 ms following the onset of the pitch shift and lasting at least 50 ms^69^. The magnitude of a vocal response was measured in cents by subtracting the mean of the baseline period (−200 to 0 ms) from the peak value of the voice contour following the response onset. The response latency was defined as the time when the response exceeded 2 SDs above or below the baseline period following the onset of the pitch shift. In addition, the SD of the baseline mean F_0_ for the averaged response was measured as an index of the amount of variability in the vocalization without feedback perturbations.

### EEG data acquisition and analyses

A 64-electrode Geodesic Sensor Net (Electrical Geodesics Inc.) was used for the acquisition of the EEG data. The impedances of individual sensors were adjusted and maintained below 50 kΩ throughout the recording^70^. The EEG signals were amplified by a Net Amps 300 amplifier (Electrical Geodesics Inc.) and recorded onto a Mac Pro computer using NetStation software (v.4.5, Electrical Geodesics Inc.). During the online recording, the EEG signals from all channels were referenced to the vertex (Cz) and digitized at a sampling frequency of 1 kHz.

After data acquisition, the EEG signals were analyzed off-line using NetStation software. Data were band-passed filtered (1–20 Hz) and segmented into epochs from −200 ms to +500 ms relative to the onset of the pitch shift. Artifact detection was performed to detect trials contaminated by excessive muscular activity, eye blinks, or eye movements. Any segment with voltage values exceeding ±55 μv of the moving average over an 80-ms window was rejected from further analyses. Additional visual inspection of all individual trials was then completed to ensure that artifacts were rejected appropriately. Individual electrodes were rejected if they contained artifacts in more than 20% of the segments, and the file was excluded if it contained more than 10 bad channels. On average, 81% of trials were retained for each condition. All channels were re-referenced to the average of electrodes on each mastoid, and artifact-free trials were averaged and baseline-corrected to generate an overall response across each condition. Since the cortical responses to pitch-shifted voice auditory feedback are mostly pronounced in the N1-P2 complex^29^,^71^, the amplitudes and latencies of N1 and P2 components were measured as the negative and positive peaks in the time windows of 80–180 ms and 160–280 ms after the onset of the pitch shift.

### Graphical network analysis

A graph is a topographical representation of a network that consists of nodes (vertices) and connections between these nodes (edges), which is generally characterized by a clustering coefficient *C* and a path length *L*. The clustering coefficient *C* is a measure of the tendency of network elements to form local clusters, while the path length *L* is a measure of parallel information transfer or global efficiency of a network^72^. In the present study, the graphical network analysis was performed using HERMES software^73^ on the EEG data in the theta band (3–8 Hz), since the spectro-temporal dynamics of ERP responses to pitch feedback perturbations is primarily pronounced in this band^74^.

The first step in applying graphical network analysis is to evaluate the correlation between all pair-wise combinations of EEG channels by computing a synchronization matrix with the synchronization likelihood (SL). Details about the calculation of SL can be found elsewhere^75^, and we give a brief description here. The SL is a measure for detecting linear and nonlinear interdependencies between two time series *X* and *Y*. Before calculating the SL, we need to convert the time series recorded from *X* and *Y* as a series of state space vectors^76^:
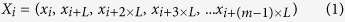

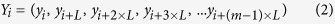
where *L* is the time lag and the *m* the embedding dimension. The SL between *X* and *Y* can be formally defined as:
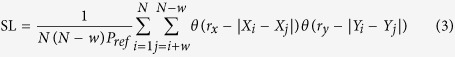
where *r*_*x*_ and *r*_*y*_ denote the cutoff distances. The SL ranges between *P*_*ref*_ (a number close to 0) and 1. *P*_*ref*_ is a parameter that reflects the small but nonzero likelihood of coincident pattern recurrence in the case of independent time series, which was set at 0.01 in present study. The SL equals to 1 in the case of the maximal synchronization of all the time series. In present study, the SL was calculated between each pair of electrodes, resulted in a square N × N matrix of size 64 (the number of EEG channels) per subject for theta EEG band.

The next step is to characterize the graph in terms of the clustering coefficient *C* and the path length *L*. A N × N (N = 64) binary graph, *G*, consisting of nodes and undirected edges, was constructed from the SL matrix of all channels by applying a correlation threshold *T* to the partial correlation coefficients:
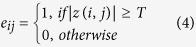


That is, an edge exists only when the absolute *z(i, j)* exceeds a threshold *T*. The subgraph *G*_*i*_ is defined as the set of nodes that are the direct neighbors of the *i*th node. As a measure to evaluate the degree of sparsity of a network, the degree of connectivity *K* is defined as the average of the degrees of all the nodes *k*_*i*_ in the subgraph *G*_*i*_:
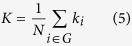


The clustering coefficient *c*_*i*_ of a node *i* is defined as^77^:
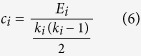
where *E*_*i*_ is the number of existing connections among the neighbors of node *i*. The denominator term *k*_*i*_(*k*_*i*_−1)/2 quantifies the number of all possible connections among the neighboring nodes. *c*_*i*_ was set to 0 when a node *i* had only one edge or no edges. The clustering coefficient *C* of a network is then defined as the mean clustering coefficient over all nodes in the network:
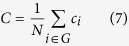


The shortest absolute path length *l*_*i*_ of a node *i* is defined as^77^:
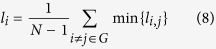
where min {*l*_*i, j*_} is the shortest absolute path length between the *i*th node and the *j*th node. The mean shortest absolute path length *L* of a network was measured as the mean minimum number of edges that link any two nodes in the network. In order to overcome the problem of dramatically increased *L* values caused by nodal pairs that have no connecting path, *L* was measured using the harmonic mean distance^78^:
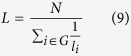


As compared to random networks, the small world is characterized by a higher degree of clustering coefficient (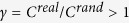) but similar path length (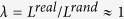) in individual network nodes^77^. The small world properties can also be examined by a measure of small-worldness, *σ* = *γ*/λ, which is typically > 1 for small-world networks^76^.

When calculating *C* and *L* as a function of threshold *T*, the topological properties of the resulting networks might be influenced by a difference in the mean level of synchronization between patients with TLE and healthy controls. To control for this effect, we calculated *C* and *L* of each brain functional network as a function of degree *K*, ensuring that graphs in both groups have the same number of edges and that the differences in *C* and *L* between two groups reflect differences in graph organization^76^.

The values of *C*^*real*^ and *L*^*real*^ need to be compared with the values of random networks as a function of degree *K* to examine the small-world properties. The values of two measures for random networks were calculated as^76^:
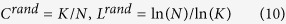


However, statistical comparisons should generally be between networks that have equal or similar degree sequences. Because the theoretical random networks have Gaussian degree distributions that may differ from the degree distribution of the brain networks for participants in the present study, we generated 30 random networks for each degree *K* in each individual network using a Markov-chain algorithm^79^,^80^. This procedure was repeated until the topological structure of the original matrix was randomized^81^, and we averaged all generated random networks to obtain a mean *C*^*rand*^ and a mean *L*^*rand*^ for each degree *K*. Finally, for the degree *K* (9 ≤ *K* ≤ 14) with increments of steps of 0.25, the topological indices of ordered, random, and real experimental networks were computed.

### Statistical analyses

The magnitudes and latencies of vocal responses and the N1-P2 complex were subjected to repeated-measures analyses of variance (RM-ANOVAs) in SPSS (v.16.0). Vocal responses were analyzed using two-way RM-ANOVAs, in which stimulus magnitude (+50 vs. +200 cents) was chosen as a within-subject factor and group (patients with TLE vs. healthy controls) as a between-subject factor. The amplitudes and latencies of the N1-P2 complex were subjected to three-way RM-ANOVAs, including within-subject factors of stimulus magnitude, and electrode site (FC1, FC2, FCz, FC3, FC4, C1, C2, Cz, C3, C4) and a between-subject factor of group. Frontal and central electrodes were chosen for statistical analyses because cortical responses to pitch-shifted voice auditory feedback are most pronounced in these electrodes^29^,^82^. Significant higher-order interactions between conditions caused subsidiary RM-ANOVAs. Probability values were corrected using Greenhouse-Geisser and corrected *p* values were reported along with original degrees of freedom when the assumption of sphericity was violated. In addition, statistical comparisons of *C*, *L*, *λ*, and *γ* as a function of group and stimulus magnitude were performed by using a two-sample two-tailed t-test for each value over a wide range of *K*. *P*-values that were smaller than 0.05 were considered significant.

## Additional Information

**How to cite this article**: Li, W. *et al*. Temporal Lobe Epilepsy Alters Auditory-motor Integration For Voice Control. *Sci. Rep.* **6**, 28909; doi: 10.1038/srep28909 (2016).
